# Pembrolizumab versus Pembrolizumab plus Chemotherapy in Non-small Cell Lung Cancer with High PD-L1 Expression - Multicenter Real-world Evidence Study

**DOI:** 10.7150/jca.113815

**Published:** 2025-07-01

**Authors:** Martin Svaton, Magdalena Knetki-Wroblewska, Petr Hosek, Gabriela Chowaniecova, Jan Spacek, Ondrej Fischer, Ondrej Bilek, Michal Hrnciarik, Diego Kauffmann-Guerrero

**Affiliations:** 1 Department of Pneumology and Phthisiology, Charles University, Faculty of Medicine in Pilsen, University Hospital in Pilsen, Pilsen, Czech Republic.; 2 Department of Lung Cancer and Chest Tumours Maria Sklodowska-Curie National Research Institute of Cancer, Warsaw, Poland.; 3 Laboratory of Cancer Treatment and Tissue Regeneration, Biomedical Center, Faculty of Medicine Pilsen, Charles University, Pilsen, Czech Republic.; 4 Department of Clinical Oncology, Specialised Hospital of St Zoerardus Zobor, Nitra, Slovakia.; 5 Department of oncology, First Faculty of Medicine, Charles University and General University Hospital in Prague, Prague, Czech Republic.; 6 Department of Respiratory Medicine, University Hospital Olomouc and Faculty of Medicine and Dentistry, Palacky University, Olomouc, Czech Republic.; 7 Department of Oncology, Masaryk Institute of Oncology, Brno, Czech Republic.; 8 Pulmonary Department, University Hospital Hradec Kralove and Faculty of Medicine in Hradec Kralove, Charles University, Hradec Kralove, Czech Republic.; 9 Department of Medicine V, University Hospital, LMU Munich, Member of the German Center for Lung Research (DZL), Munich, Germany.

**Keywords:** NSCLC, immunochemotherapy, pembrolizumab, chemotherapy, high PD-L1 expression, real-world evidence

## Abstract

**Background:** Patients with non-small cell lung cancer (NSCLC) with PD-L1 expression ≥ 50% can be treated with immunotherapy alone or with a combination of immunotherapy and chemotherapy. One of these options is treatment with pembrolizumab (P) with/without chemotherapy (CHT). Meta-analyses from randomized trials suggest a beneficial effect on response rate (RR) or progression free survival (PFS) when using the combination treatment P + CHT compared to P alone, but not on improving overall survival (OS). However, data from real-world clinical practice are insufficient especially in European patients. Regional differences, e.g. in the representation of KRAS mutations between Asian and European patients, could theoretically influence potential differences between P + CHT and P. Therefore, the aim of this study was to compare P + CHT versus P alone in real clinical practice in patients from Central Europe.

**Methods:** Retrospective data from 8 comprehensive oncology centres in Central Europe were used. All patients with PD-L1 expression ≥ 50% with stage IV NSCLC treated with pembrolizumab in daily practice to June 2024 were included and their data statistically analysed.

**Results:** In the whole group 793 patients was included in the study - 706 treated with P and 87 with P+ CHT. In this unadjusted sample, we observed significantly higher RR (p <0.0001) and OS (p = 0.044) for the P + CHT group vs. P. For significant differences in both groups, where performance status in particular played a role in survival in the Cox model, we subsequently performed patient matching 2 (P+CHT):1 (P) from the whole group of patients. After this patient matching, we continued to observe a significant difference in RR (p = 0.005), but no longer in OS (p = 0.103). The PFS was not significantly different in both cases (p= 0.174 for unadjusted patients resp. p = 0.342 for matching groups).

**Conclusions:** P+CHT leads to a significantly higher RR compared to P and can therefore be considered in patients with a more certain treatment response goal (e.g., bulky symptomatic tumor), however, this advantage does not translate into PFS and OS benefit.

## Introduction

Immunotherapy with possible chemotherapy is the gold standard of treatment for patients with non-small cell lung cancer (NSCLC) with high PD-L1 expression (PD-L1 ≥ 50%) 1. One of the most commonly used treatment modalities for these patients is pembrolizumab (P) with a possible addition of chemotherapy (CHT). Pembrolizumab monotherapy demonstrated superiority over chemotherapy in high PD-L1 expressors with NSCLC in the phase III clinical trial KEYNOTE-024 2. Pembrolizumab combined with chemotherapy then demonstrated a benefit over chemotherapy in the phase III clinical trials KEYNOTE-189 (for nonsqamous NSCLC) and KEYNOTE-407 (for squamous NSCLC), including patients with high PD-L1 expression 3, 4. P monotherapy brings long-term survival to only a minority of patients 2. Therefore, it is questionable whether the addition of CHT can bring any benefit to patients with NSCLC with high expression of PD-L1 over the use of P alone.

Meta-analyses of randomized clinical trials mostly showed a higher objective response rate (ORR) and progression-free survival (PFS) with no improvement in overall survival (OS) and increased adverse effects when using P+ CHT versus P 5, 6. However, clinical trials include a selected group of patients and do not fully reflect real clinical practice 7. Data from real world evidence (RWE) are limited - especially for European patients 8-12. Therefore, the aim of this multicentric international retrospective study was therefore to compare P treatment with P + CHT in real clinical practice in a European population.

## Materials and Methods

### Study design and treatment

Retrospective data from 5 Czech (Brno, Hradec Kralove, Olomouc, Pilsen and Prague), 1 Polish (Warsaw), 1 German (Munich) and 1 Slovakia (Nitra) comprehensive oncology centres were used. All patients with stage IV NSCLC and PD-L1 expression ≥ 50% treated at the individual centres as part of routine clinical practice with first line P +/- CHT until June 2024 (since the start of use of this treatment in the centres) were included. All patients were treated according to the current clinical guidelines using first line pembrolizumab at a dose of 200mg q3w or 400mg q6w intravenously or pembrolizumab (in same doses) with platinum chemotherapy doublet (for 4 cycles, pemetrexed as maintenance therapy was possible). Clinical follow-ups including physical examination, plain chest X-ray and routine laboratory tests were performed every 3 or 6 weeks. CT scans were performed every 3 to 4 months. The purpose of the study was to compare the response rate (RR), progression-free survival (PFS) and overall survival (OS) between the pembrolizumab monotherapy arm and the pembrolizumab combined with chemotherapy arm in the overall population. Moreover, we also have analysed survival parameters in subgroups divided according to the PD-L1 expression with cut-off 80 % based on the article by Frost *et al.* 13.

### Statistics

Standard frequency tables and descriptive statistics were used to characterize the patient samples. RR was defined according to the RECIST 1.1 criteria 14. PFS was defined as the time from the initiation of treatment to the progression of the disease or to death from any cause. OS was defined as the date from the initiation of treatment to death from any cause. Living patients were censored on the last date on which they were known to be alive. Furthermore, a few patients were censored on the date of loss for follow-up.

First, the patients receiving P + CHT treatment were compared to all patients in the sample receiving P treatment only with respect to their basic clinical characteristics to assess the comparability of these groups (using appropriate tests as indicated in Table 1). Then, the differences between these groups in RR were evaluated both ordinally, using the Mann-Whitney U test, and categorically, using Fisher's exact test. Both methods provided qualitatively identical results. Subsequently, the analysis of PFS and OS in these groups was performed using the Kaplan-Meier method with the Gehan-Wilcoxon significance test as well as a multifactorial categorical Cox regression model to assess the prognostic independence of therapy type on common clinical factors (i.e. smoking, sex, Eastern Cooperative Oncology Group performance status = ECOG PS, age, histology, stage and PD-L1 expression).

Due to considerable clinical differences between the P + CHT group and the whole-sample P group, we decided to repeat the analysis using a matched sample. The matched sample was assembled using all patients from the P + CHT group for whom all the required matching variables were available (N = 84). For each of these patients, 2 closest-matching counterparts receiving P only were selected without repetition based on sex, ECOG PS, age, histology, stage and PD-L1 expression. The matching was performed using a proprietary script in MATLAB (R2024b, The MathWorks, Inc., Natick, MA, USA) based on Euler distances in normalized variable space, first selecting one counterpart for each patient and then selecting the second one for each in the same order. The whole process was repeated at least a thousand times using randomized assignment order and the result providing the best overall match between the groups was used as the final matched sample. The analysis of RR, PFS and OS was then repeated in the matched sample using the same methods.

The median duration of follow-up was estimated using the inverse Kaplan-Meier method. The analysis was performed in Statistica (version 10Cz, StatSoft, Inc., Tulsa, OK, USA). The level of statistical significance was established at α = 0.05 and all reported p-values are two-tailed.

## Results

### Patient characteristics

A total of 793 patients were included in the study. 706 (89%) were treated with P and 87 (11%) with P + CHT. In the entire sample of patients, the median age was 69 years (range: 37 - 90 years) and the median of PD-L1 expression was 80% (50-100%). In the P group, the median age was 69.7 years (37-90 years) and the median of PD-L1 expression was 80% (50-100%). In the P + CHT group, the median age was 65 years (39-81 years) and the median of PD-L1 expression was 70% (50-100%). In the P + CHT group, the chemotherapy part was represented as follows: pemetrexed + cisplatin 1 patient (1.2 %), pemetrexed + carboplatin 63 patients (72.4 %), paclitaxel + carboplatin 21 patients (24.1 %) and docetaxel + carboplatin 2 patients (2.3 %). Other patient characteristics of the whole cohort of patients (also including differences between P and P + CHT arms) are summarized in Table 1. The median follow-up time reached 29.4 months.

### Response rate (RR) in unadjusted sample

**Figure 1:**
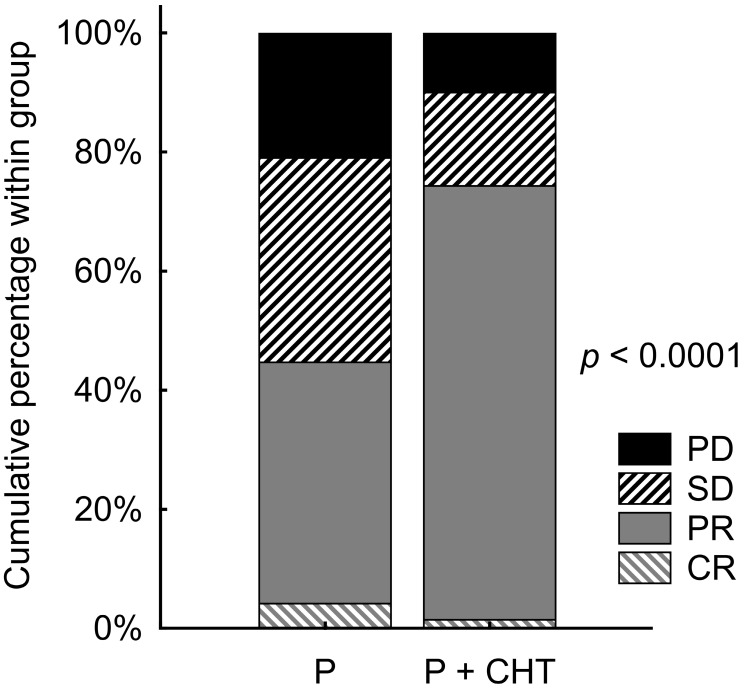
Response rate in unadjusted patients (i.e. all patients sample).

The objective RR was known in our database for 646 patients (576 in the P group and 70 in the P + CHT group). Patients with P + CHT had significantly better RR than those with P alone (p <0.0001). Objective RR (ORR) reached 44.6% of patients in the P group and 74.3% of patients in the P + CHT group. Details are shown in Table 2 and Figure 1.

### PFS and OS in unadjusted sample

**Figure 2:**
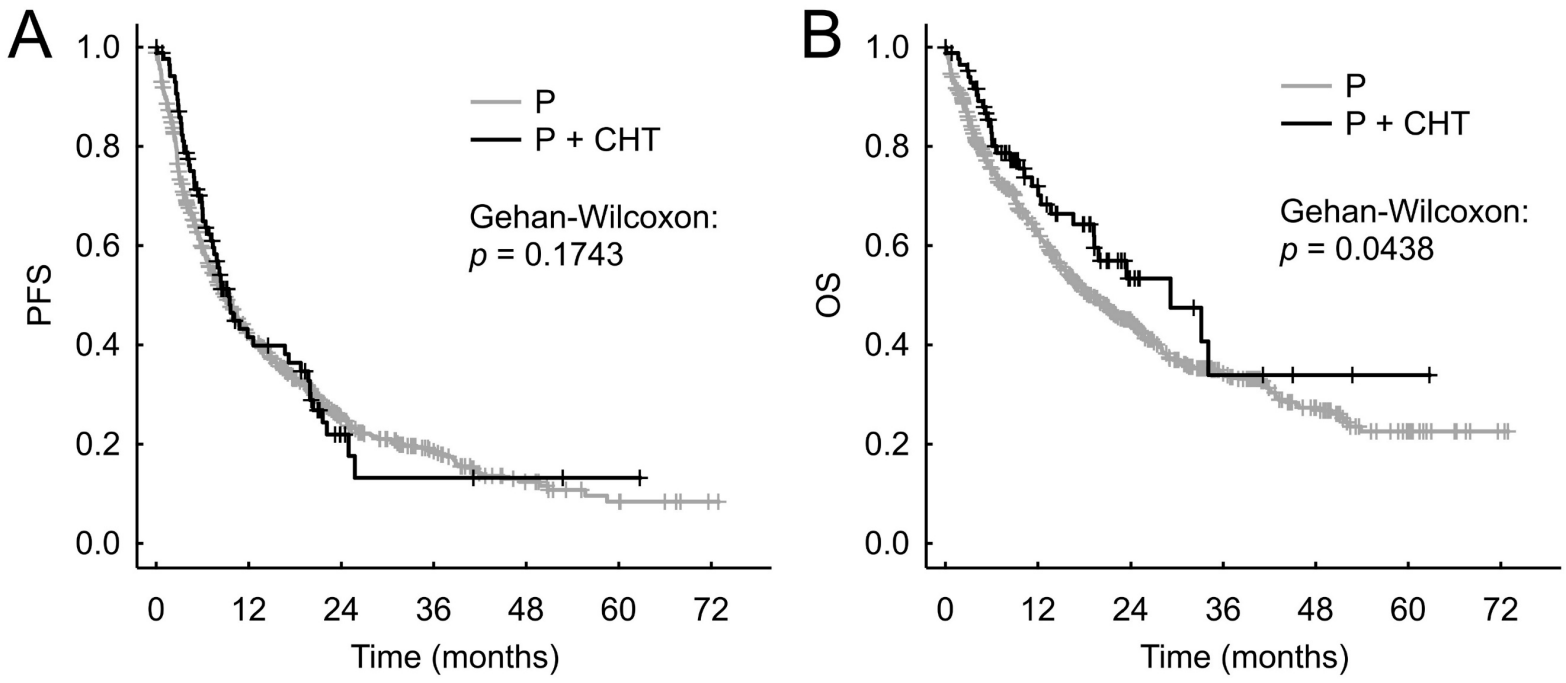
Progression free survival (A) and Overall survival (B) for unadjusted patients (i.e. all patients sample).

We did not observe statistically significantly different PFS between the P+CHT and P groups in the entire cohort of patients - the median PFS were 9.2 months (95% CI 7.2-15.6 months) vs 8.9 months (95% CI 7.7-10.3 months), respectively (p = 0.174, Figure 2A).

The P+CHT group showed significantly better OS than the P group in entire cohort of patients - median OS were 26.8 months (95% CI 19.1 months-N.A.) compared to 18.5 months (95% CI 16.2-21.8 months), respectively (p = 0.044, Figure 2B).

### Cox regression model for unadjusted sample

The only significant factor that affected both PFS and OS in the Cox multivariate model was ECOG PS. Other clinical factors - including high vs. low PD-L1 expression, histology and treatment type (P vs. P + CHT) did not significantly affect PFS or OS. Further details are shown in Table 3.

### RR, PFS and OS for matched patients

84 patients with fully known characteristics from the P + CHT group were assigned 168 patients (matching 1:2) from the P group with the most similar patient characteristics (patients characteristics are shown in Table 4) - i.e. patients chosen from the whole group of 793 patients.

**Figure 3:**
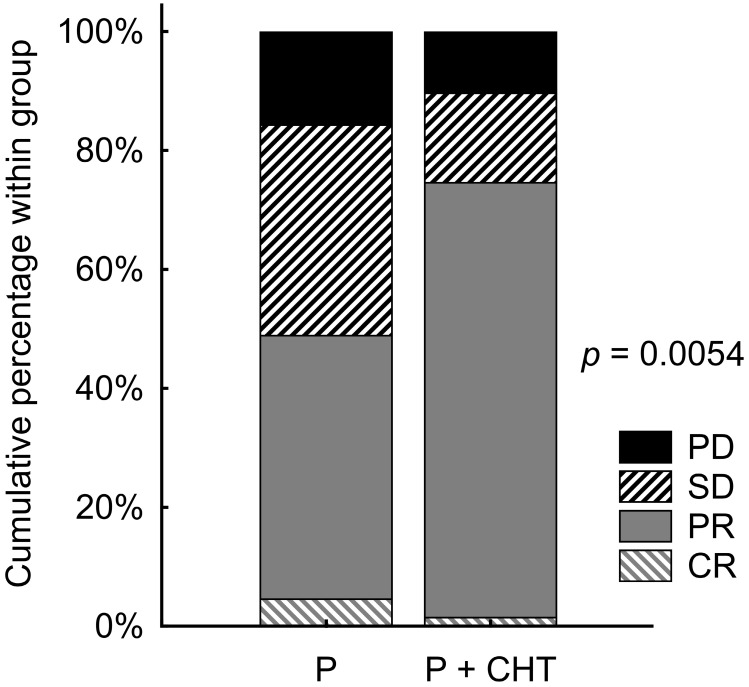
Response rate in matched patients (selected from the whole group of patients).

The objective RR remained significantly better for the group of patients treated with P + CHT vs. P (p = 0.005). Objective RR (ORR) reached 48.9% of patients in the P group and 74.6% of patients in the P + CHT group. Details are shown in Figure 3.

**Figure 4:**
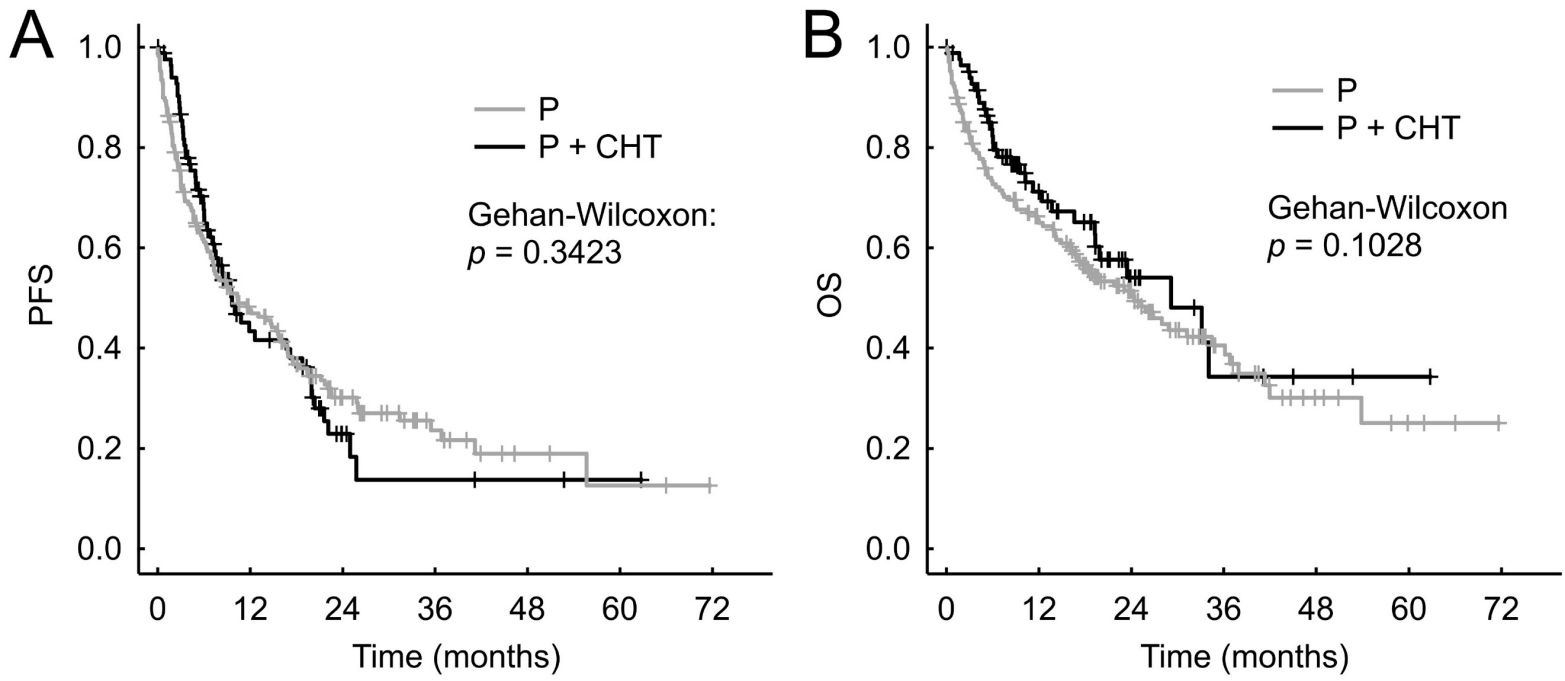
Progression free survival (A) and Overall survival (B) in matched patients (selected from the whole group of patients).

We did not observe statistically significantly different PFS between the P and P + CHT groups - the median PFS was 10.3 months (95% CI 7.0-15.7 months) vs. 9.5 months (95% CI 7.1-17.1 months), respectively (p = 0.342, Figure 4A).

Similarly, we did not observe a statistically significant difference in OS between the P and P + CHT groups - median OS was 24.3 months (95% CI 17.0-33.7 months) vs 27.3 months (95% CI 19.3-N.A. months), respectively (p = 0.103, Figure 4B).

## Discussion

Our multicenter international retrospective real-world clinical study demonstrated a significant improvement in RR in patients treated with P + CHT vs. P alone in a sample of European patients with stage IV NSCLC and high PD-L1 expression where RWE data were insufficient and, for example, due to different mutation rates, they could differ from other regions. The P+CHT group showed significantly better OS than the P group in the entire cohort of patients, however, after matching - there were no differences in survival parameters between subgroups.

Meta-analyses comparing immunotherapy alone vs. immunochemotherapy in NSCLC with high PD-L1 expression showed better RR and PFS for combination therapy, but no significant improvement in OS 15-18. The improvement in PFS and RR came at the cost of higher toxicity of treatment associated with chemotherapy 15. The meta-analysis by Wang *et al.* points to possible differences according to histology 16. However, these studies included different drugs and did not focus solely on comparing P versus P+CHT. However, similar results were also shown by meta-analyses comparing P alone vs. P + CHT, where the combination treatment resulted in better RR and PFS with higher toxicity. OS was not significantly prolonged (although a certain trend could be observed in some meta-analyses) 5, 6, 19, 20. The results for PFS are inconsistent with our data. However, in clinical randomized trials, there is some selection of patients (e.g., from the side of the allowable ECOG PS in contrast to our study, where also ECOG PS 2 patients were included) and therefore they do not fully reflect real clinical practice.

Our results also show the difference between real clinical practice and the clinical trials. While PFS was comparable to clinical trials in the P arm, OS was lower 21, 22. This could be due to the poorer condition of patients in our study, who are no longer able to start second-line treatment after progression. Comparing our data for P + CHT with clinical trials is difficult, as these studies with pembrolizumab were conducted differently with respect to NSCLC histology, and in addition, the group with PD-L1 expression ≥ 50% was only a subgroup of the total sample in these studies 3, 4.

RWE studies comparing immunotherapy and chemoimmunotherapy in NSCLC with PD-L1 ≥ 50% are relatively rare. McLouth *et al.* compared the quality of life between patients treated with immunotherapy vs. chemoimmunotherapy in a smaller study (n = 60) 23. Except for the decreased in appetite during chemoimmunotherapy, they did not find any differences in the quality of life of the patients. Takumida *et al.* observed better RR and PFS in patients treated with P + CHT vs. P in 126 Asian patients 10. This study did not compare OS. However, it is known that Asian populations have a different genetic profile (frequency of KRAS mutations in particular with respect to immunotherapy) compared to European populations 24. This may explain the different finding with respect to PFS. Shah *et al.* compared immunotherapy with chemoimmunotherapy in NSCLC patients with PD-L1 expression ≥ 50% using retrospective US data 8. In a cohort of 3086 patients, they did not observe significantly higher OS inpatients treated with chemoimmunotherapy overall. However, they did note a better OS during the first six months of treatment. This phenomenon could be related to the prevention of hyperprogression (which can affect up to 14% of patients with NSCLC) in patients treated with chemoimmunotherapy 8, 25. This would also be suggested by the meta-analysis indicating a lower number of early-progressing patients when chemoimmunotherapy was used 17. The reduction in the risk of hyperprogression could be reflected in the higher RR of P + CHT, when chemotherapy acts synergistically with immunotherapy 16, 26. Since the combination therapy may yield a higher RR but also higher treatment toxicity, it might be necessary to define suitable candidates for this treatment modality. Morimoto *et al.* showed higher RR in the case of chemoimmunotherapy vs. pembrolizumab monotherapy applied only to patients with good PS and not to patients with higher PS 27. This could be explained by the prevalence of increased toxicity in this group of patients with poorer completion of the planned treatment. This is also shown by data for older patients, where both German and Japanese studies in older patients did not show an improvement in prognosis for combination therapy 11, 28. On the contrary, chemoimmunotherapy in older patients led to a higher number of adverse events associated with a greater number of hospitalizations and a higher rate of treatment discontinuation 11, 28. In patients with high disease burden or those with aggressively growing tumors, combination therapy might prevent hyperprogression and thus increase the chances of further treatment 8, 25, 29. However, our study after matching patients did not confirm that such higher RR would translate into a better prognosis for these patients. The better OS in the unadjusted sample was most likely achieved due to the greater representation of patients with ECOG PS 0 in the combination treatment arm, with ECOG PS being the only independent factor for both PFS and OS in the Cox model. As shown by our Kaplan-Meier curves, the trend for better PFS and OS for the combination of P + CHT was seen at the beginning of treatment, in accordance with the US data, but it did not translate into the overall prognosis of the patients, in agreement with these authors 8. In the Cox model, we did not observe significant associations with age or high/low PD-L1 expression. The only significant factor was ECOG PS. The question then is whether in highly selected patients with a theoretical risk of hyperprogression (e.g. high disease burden or aggressively growing tumors) and good ECOG PS, the combination of P + CHT based on a higher RR is actually beneficial when this advantage does not clearly translate into a better prognosis for these patients. On the other hand, in patients with high disease dynamics and symptoms such as dyspnea or cough, faster tumor shrinkage has the potential to translate into improved quality of life. This is an important goal of palliative systemic therapy.

This study has several limitations. This is a retrospective study and thus the selection criteria for the treatment choice for specific patients might differ over time and by center and therefore, general criteria for choosing P or P + CHT treatment cannot be stated. At the same time, the retrospective design led to some imbalances between the treatment arms - there were especially more patients with ECOG PS 0, stage IVB and adenocarcinomas in the P + CHT arm. While the difference in the representation of histological subgroups may be due to reimbursement conditions (in the Czech Republic, combination therapy is not reimbursed for squamous cell carcinomas with high PD-L1 expression), the better ECOG PS and higher number of stages IVB patients may reflect the tendency to administer the combination therapy to patients who are more fit but have a larger tumor burden. The difference in ECOG PS then turned out to be the only significant one for both PFS and OS in our Cox model, and the differences in the representation of patients according ECOG PS in the unadjusted groups most likely caused a bias for a significantly higher OS in the unadjusted sample. Therefore, we tried to remove these imbalances between patient groups by subsequent matching of patients, which then no longer showed a significant difference for OS. However, we then continued to confirm a significant difference for RR.

## Conclusion

Data, including ours from Europe, suggest that P + CHT significantly improves RR compared to P alone in patients with stage IV NSCLC and PD-L1 expression ≥ 50%. Although OS appeared to be longer in the P + CHT group in our unadjusted sample, this was not demonstrated after patient matching. This fact was probably due to differences between groups in the unadjusted sample, especially in ECOG PS, which significantly affected both PFS and OS in the Cox model. The use of the combination of P + CHT can therefore be considered in the case of effort for a higher chance of reducing the tumor mass (e.g. bulky symptomatic tumor), however, this treatment does not probably lead to a better prognosis for patients compared to P alone. The ongoing phase III trials LAPLACE-50 and PERSEE comparing P vs. P + CHT in NSCLC may shed further light on questions that remain unanswered at present 30, 31.

## Figures and Tables

**Table 1:** Patient characteristics (all sample).

Parameter	Category	P n (%) (n = 706)	P + CHT n (%) (n = 87)	p-value for difference between groups (test method)
Sex	Male	427 (60.5)	58 (66.7)	0.2641(Chi-squared)
	Female	279 (39.5	29 (33.3)	
ECOG PS	0	47 (6.7)	22 (25.3)	0.0004(Mann-Whitney U)
	1	641 (90.9)	58 (66.7)	
	2	17 (2.4)	7 (8.0)	
	Unknown*	1	0	
Smoking	Smoker	204 (57.1)	33 (40.7)	0.0238(Fisher)
	Exsmoker	121 (33.9)	39 (48.1)	
	Never smoker	32 (9.0)	9 (11.1)	
	Unknown*	349	6	
Stage	IIIA	2 (0.3)	1 (1.1)	<0.0001 (Mann-Whitney U)
	IIIB	3 (0.4)	2 (2.3)	
	IIIC	3 (0.4)	0 (0.0)	
	IVA	323 (45.8)	17 (19.6)	
	IVB	309 (43.8)	66 (75.9)	
	IV (without specification)**	66 (9.3)	1 (1.1)	
Histology	Adenocarcinoma	413 (58.5)	68 (78.2)	0.0003(Chi-squared)
	Sqamous carcinoma	244 (34.6)	14 (16.1)	
	Adenosqamous carcinoma**	8 (1.1)	2 (2.3)	
	Large cell carcinoma**	4 (0.6)	0 (0.0)	
	NOS**	37 (5.2)	3 (3.4)	
PD-L1 Expression	< 80%	305 (43.3)	48 (56.5)	0.0207(Chi-squared)
	≥ 80%	400 (56.7)	37 (43.5)	
	Unknown*	1	2	

* - Category not included in the percentage calculation and significance test ** - Category not included in the significance test ECOG PS = Eastern Cooperative Oncology Group performance status; NOS = not otherwise specified; P = pembrolizumab; P + CHT = pembrolizumab + chemotherapy

**Table 2:** Response rate in unadjusted patients (i.e. all patients sample).

Category	P n (%)	P + CHT n (%)
CR	24 (4.2)	1 (1.4)
PR	233 (40.4)	51 (72.9)
SD	198 (34.4)	11 (15.7)
PD	121 (21.0)	7 (10.0)
Unknown	130	17

CR = complete response; P = pembrolizumab; P + CHT = pembrolizumab + chemotherapy; PD = progressive disease; PR = partial response; SD = stable disease

**Table 3:** Cox regression model in unadjusted patients (i.e. all patients sample).

		PFS		OS	
**Factor**	**Category**	**HR (95% CI)**	**p value**	**HR (95% CI)**	**p value**
**Smoking**	No	1		1	
	Exsmoker	0.73 (0.48 - 1.12)	0.154	0.79 (0.45 - 1.35)	0.373
	Yes	0.88 (0.59 - 1.32)	0.537	1.08 (0.65 - 1.80)	0.754
**Sex**	Male	1		1	
	Female	1.06 (0.83 - 1.36)	0.640	0.81 (0.60 - 1.11)	0.190
**ECOG PS**	0	1		1	
	1	1.95 (1.26 - 3.02)	**0.003**	2.68 (1.34 - 5.14)	**0.003**
	2	3.41 (1.75 - 6.64)	**<0.001**	5.27 (2.22 - 12.54)	**<0.001**
**Age**	<70	1		1	
	≥70	0.93 (0.73 - 1.18)	0.535	0.96 (0.72 - 1.29)	0.802
**Histology**	Adenocarcinoma	1		1	
	Sqamous	1.03 (0.79 - 1.35)	0.838	0.97 (0.70 - 1.34)	0.852
	Other	1.00 (0.59 - 1.70)	0.992	1.24 (0.69 - 2.25)	0.473
**Stage**	3	1		1	
	4	1.18 (0.61 - 2.32)	0.622	1.18 (0.48 -2.90)	0.712
**PD-L1 Expression**	<80 %	1		1	
	≥80 %	0.89 (0.70 - 1.14)	0.354	0.98 (0.73 - 1.32)	0.914
**Therapy**	P	1		1	
	P+CHT	1.06 (0.76 - 1.46)	0.746	1.07 (0.71 - 1.62)	0.741

ECOG PS = Eastern Cooperative Oncology Group performance status; HR = hazard ratio; OS = overall survival; P = pembrolizumab; P + CHT = pembrolizumab + chemotherapy; PFS = progression free survival

**Table 4:** Patient characteristics (matched patients - selected from the whole group of patients)

Parameter	Category	P MATCHED n (%)	P + CHT MATCHED n (%)	p-value for difference between groups (test method)
Sex	Male	104 (61.9)	56 (66.7)	0.459(Chi-squared)
	Female	64 (38.1)	28 (33.3)	
ECOG PS	0	42 (25.0)	22 (26.2)	0.917(Mann-Whitney U)
	1	116 (69.1)	55 (65.5)	
	2	10 (6.0)	7 (8.3)	
	Unknown*	1	0	
Smoking	Smoker	46 (51.1)	31 (39.7)	0.219(Fisher)
	Exsmoker	39 (43.3)	38 (48.7)	
	Never smoker	5 (5.6)	9 (11.5)	
	Unknown*	78	6	
Stage	IIIA	2 (1.2)	1 (1.2)	1.000***(Fisher)
	IIIB	3 (1.8)	2 (2.4)	
	IIIC	1 (0.6)	0 (0.0)	
	IVA	61 (36.3)	17 (20.2)	
	IVB	82 (48.8)	63 (75.0)	
	IV (without specification)**	19 (11.3)	1 (1.2)	
Histology	Adenocarcinoma	134 (79.8)	67 (79.8)	1.000(Chi-squared)
	Sqamous carcinoma	28 (16.7)	14 (16.7)	
	Adenosqamous carcinoma**	2 (1.2)	0 (0.0)	
	Large cell carcinoma**	0 (0.0)	0 (0.0)	
	NOS**	4 (2.4)	3 (3.6)	
PD-L1 Expression	< 80%	82 (48.8)	47 (56.0)	0.285(Chi-squared)
	≥ 80%	86 (51.2)	37 (44.1)	
	Unknown*	0		

* - Category not included in the percentage calculation and significance test ** - Category not included in the significance test *** - Test only between Stage III and Stage IV ECOG PS = Eastern Cooperative Oncology Group performance status; NOS = not otherwise specified; P = pembrolizumab; P + CHT = pembrolizumab + chemotherapy

## Abbreviations

ECOG PS: Eastern Cooperative Oncology Group performance status
CHT: Chemotherapy
NSCLC: Non-small cell lung cancer
ORR: Objective response rate
OS: Overall survival
P: Pembrolizumab
PFS: Progression free survival
RR: Response rate
RWE: Real world evidence
